# Determinants and indicators of successful aging as a multidimensional outcome: a systematic review of longitudinal studies

**DOI:** 10.3389/fpubh.2023.1258280

**Published:** 2023-11-21

**Authors:** Caue Egea Rodrigues, Caine Lucas Grandt, Reem Abu Alwafa, Manal Badrasawi, Krasimira Aleksandrova

**Affiliations:** ^1^Department of Pharmacology and Toxicology, Institute of Pharmacy, Free University Berlin, Berlin, Germany; ^2^Department Epidemiological Methods and Etiological Research, Leibniz Institute for Prevention Research and Epidemiology–BIPS, Bremen, Germany; ^3^Faculty of Human and Health Sciences, University of Bremen, Bremen, Germany; ^4^Faculty of Agriculture, An-Najah National University, Nablus, Palestine

**Keywords:** successful aging, determinants, lifestyle factors, metabolic health, well-being, systematic review

## Abstract

**Background:**

Successful aging (SA) has been coined as a term to describe the multidimensional aspects associated with achieving optimal combination of physical and mental health along with social well-being health, mental and social well-being at older age. In recent years there has been an increased interest in understanding the role of determinants of SA, such as demographic, biological, behavioral, psychological and social factors. To synthesize the recent evidence, we conducted a systematic review of longitudinal studies on a range of determinants and indicators of SA defined as a multidimensional outcome.

**Methods:**

A systematic search of PubMed, MEDLINE and Web of Science for finding eligible papers published between August 2016 and June 2023 was conducted following the Preferred Reporting Items for a Systematic Review and Meta-Analysis (PRISMA) guidelines. The review protocol was registered in PROSPERO International Prospective Register of Systematic Reviews (Registration number: CRD42021250200). The web-based automated screening tool–Rayyan–was used for title and abstract screening. The study quality was assessed using the Quality in Prognosis Studies (QUIPS) tool.

**Results:**

A total of 3,191 records were initially identified using the predefined search strategy. Out of 289 articles selected for full text screening, 22 were found eligible and included in the review. A variety of factors have been explored in relation to SA, ranging from socio-demographic factors, nutrition, lifestyle, biological pathways, psychological health, and well-being. Overall, the results of recent studies have confirmed the role of metabolic health, adherence to healthy dietary patterns, such as the Mediterranean diet, physical activity, non-smoking, and higher socio-economic status as main factors associated with higher odds for SA. Emerging research highlights the role of psycho-social factors and early life health as determinants of SA.

**Conclusion:**

In summary, this review highlights the importance of healthy living and monitoring metabolic risk along with sustaining psychological well-being in adult life as major determinants of SA. Further methodological and research work on SA would pave the way toward development of adequate health promotion policies in aging societies.

**Systematic review registration:**

https://www.crd.york.ac.uk/prospero/display_record.php?ID=CRD42021250200, CRD42021250200.

## 1 Introduction

The world’s population is aging at an unprecedented pace. By 2050, it is estimated that the global number of people aged 65 and older will be more than double, reaching over 1.5 billion individuals (1). The marked increase in the proportion of older people has posed new challenges to public health systems and societies across the globe (2). How to cope with the increasing burden of age-related diseases and conditions has become a point of major concern to health policy makers (3). To address this question, it is not only vital to understand drivers of chronic disease development, but also determinants that ensure overall health and well-being at older age. The concept of successful aging (SA) was introduced by Rowe and Kahn in 1997 to describe the phenomenon of aging in good overall physical, mental, and social wellbeing (4). Originally defined as ‘freedom from disease or disease-disability, high cognitive and physical functioning, and active engagement with life’, the term SA has evolved to include functional ability, independence and quality of life that assure not only living longer but also preserving health and wellbeing until old age (5–7). There has been increasing interest in understanding the role of various exposures throughout the life-course including lifestyle, biological, psychological, and social factors as possible determinants of SA. Several reviews have been previously conducted to evaluate determinants of SA. However, those reviews were focused on specific factors, such as physical activity (8, 9), smoking and alcohol consumption (10), diet (11, 12), and psychosocial factors (13, 14). So far, only one review provided a comprehensive overview of a wider range of determinants including demographic, biological, behavioral, psychological and social factors summarizing evidence published by August 2016 (15). With the continuously increasing interest in the topic, numerous new studies have been published in the recent years. However, the newly emerging evidence has not been summarized yet.

We therefore conducted a systematic review to synthesize the recent evidence from published longitudinal studies on a range of determinants and indicators of SA to identify existing gaps and guide planning of future studies and the development of tailored interventions and policies addressing healthy longevity.

## 2 Methods

### 2.1 Search strategy and selection criteria

The review protocol has been developed and registered in PROSPERO International Prospective Register of Systematic Reviews (Registration number: CRD42021250200). The review was written following the Preferred Reporting Items for a Systematic Review and Meta-Analysis (PRISMA) guidelines (16). To enable full exploration of the literature on the topic, no setting restrictions were applied. A systematic search was performed identifying records in PubMed, MEDLINE, and Web of Science published between August 2016 and June 2023 in order to depict literature published after a previous review on the topic (15). The following terms were included in our search methodology: (1) outcome defining terms: “healthy aging” OR “healthy aging” OR “successful aging” OR “successful aging” OR “positive aging” OR “positive aging” OR “productive aging” OR “productive aging” OR “aging well” OR “aging well” OR “effective aging,” and (2) exposure defining terms: “smoking” OR “tobacco” OR “cigarette*” OR “physical activity” OR “physical inactivity” OR “exercise*” OR “alcohol” OR “alcohol drinking” OR “diet” OR “nutrition” OR “quality of life” OR “health” OR “physical health” OR “physiological well-being” OR “metabolic health” OR “mental health” OR “cognitive function” OR “social well-being” OR “factor*” OR “indicator*” OR “correlate*, and (3) “study type defining terms: “longitudinal” OR “cohort stud*.”

A summary of the inclusion and exclusion criteria and rationale is presented in Supplementary Table S1. Only longitudinal observational studies, where exposures were measured before the outcome, were included in the review. Further inclusion criteria included: (a) original research articles published in peer-reviewed journals; (b) primary aim to measure associations between determinants and SA outcome; (c) using the definition of SA based on the multidimensional model of SA by Rowe and Kahn, i.e., including at least some aspects within the predefined criteria: (1) absence of chronic disease and disability; (2) good physical and cognitive functioning and (3) engagement with life (sustained engagement in social and productive activities); (d) community-dwelling populations; (e) baseline age of participants being >18 years. Exclusion criteria were: (a) cross-sectional and case–control studies, experimental laboratory or animal studies, and methodological studies; (b) secondary source reports (including literature reviews, books, consensus statement, expert meetings, policy recommendations, and author opinions); (c) SA definitions not including the aforementioned domains of SA as defined by Rowe and Kahn; (d) studies in special population groups, i.e., pregnant women, diseased individuals, institutionalized individuals, and centenarians. Determinants and indicators of SA explored in this review were organized into the following four domains: (1) socio-demographic; (2) nutrition and lifestyle-related; (3) biological; and (4) psychological factors and well-being.

### 2.2 Study selection

Identified records were imported into the Rayyan, a web-based automated screening tool for systematic reviews (17). Titles and abstracts were then screened by two independent reviewers (RAA, AWES). Any conflict between the two reviewers was resolved by a third independent reviewer (CER). Full-text articles were retrieved if the article was considered eligible and subjected to a second evaluation by three independent reviewers (RAA, AWES, and CLG). Any discrepancies and disagreements in the full-text screening were discussed and resolved by consensus among three independent reviewers (CER, KA, and MB). After retrieval of full-text articles, the reference lists of selected articles were additionally cross-checked to identify further articles of relevance.

### 2.3 Data extraction

The data extraction was performed by two reviewers (AWES and CER) using a predefined data extraction form. The following information was extracted: first author, publication year and country, study design, study population, sample size, details on composition and measurement of SA outcome, details on assessed determinants, and reported effect estimates for associations with the SA outcome. When a study provided several effect-estimates with adjustment for different sets of covariates, results were reported based on the one adjusting for the largest number of covariates.

### 2.4 Assessment of risk of bias

The quality assessment of the included studies was performed by three independent reviewers (RAA, AWES, and CER) using the Quality in Prognosis Studies (QUIPS) tool. The QUIPS tool was developed to assess the risk of bias (RoB) in studies on prognostic factors (18). The specific questions and assigned points of the QUIPS tool are presented in Supplementary Tables S2–S8, respectively. The overall RoB for each study was evaluated based on the bias domains including study participation, study attrition, outcome measurement, study confounding, as well as statistical analysis and reporting. Each study was assigned an overall bias rating of low, medium, or high, based on the assessment of each separate bias domain.

## 3 Results

### 3.1 Study selection

**Figure 1:**
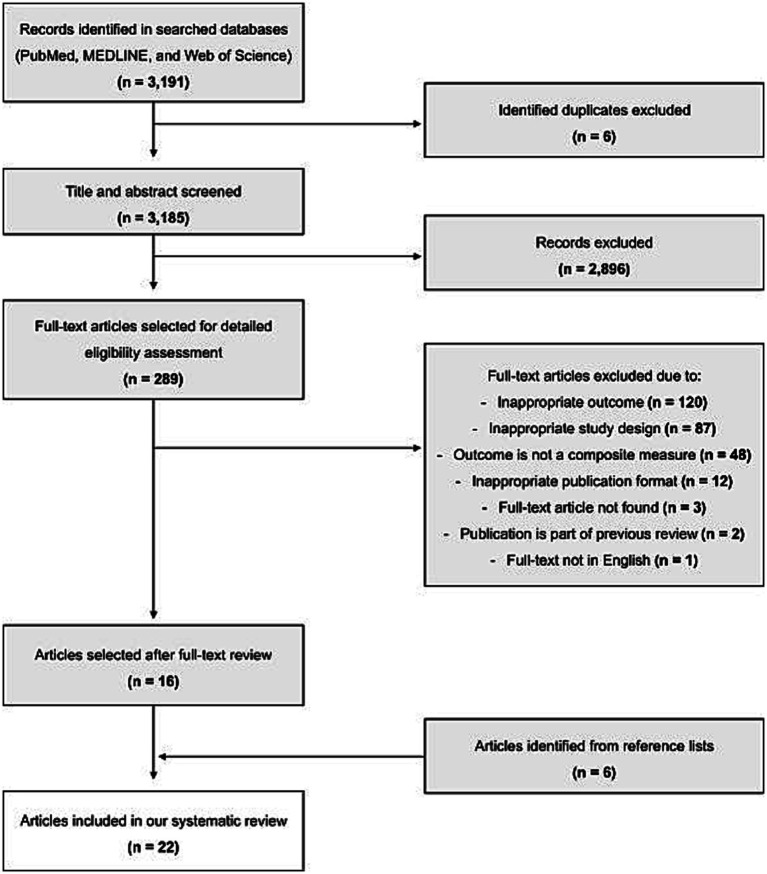
PRISMA flow diagram of study selection, including identification, screening, eligibility, and inclusion of studies.

A total of 3,191 records were initially identified in PubMed, MEDLINE, and Web of Science using the defined search strategy (Figure 1). After excluding duplicates (*n* = 6), the title and abstract of a total of 3,185 records were screened. Of these, 2,896 were deemed ineligible and 289 records remained for full-text screening. Of these, 273 articles were excluded due to inappropriate outcome (*n* = 120), inappropriate study design (*n* = 87), outcome not a composite measure of SA (*n* = 48), inappropriate publication format (*n* = 12), full-text article not found (*n* = 3), publication was part of a previous review (*n* = 2), full-text not in English (*n* = 1). Following the full-text screening, 16 articles were found eligible according to the proposed inclusion and exclusion criteria and their reference lists were screened for the identification of potential eligible studies, retrieving a total of 6 additional articles. In total, 22 articles met the selection criteria and were therefore included in the systematic review (19–40). Figure 1 shows the PRISMA flowchart with studies included and excluded at each step of the systematic review process.

### 3.2 Study characteristics

**Figure 2:**
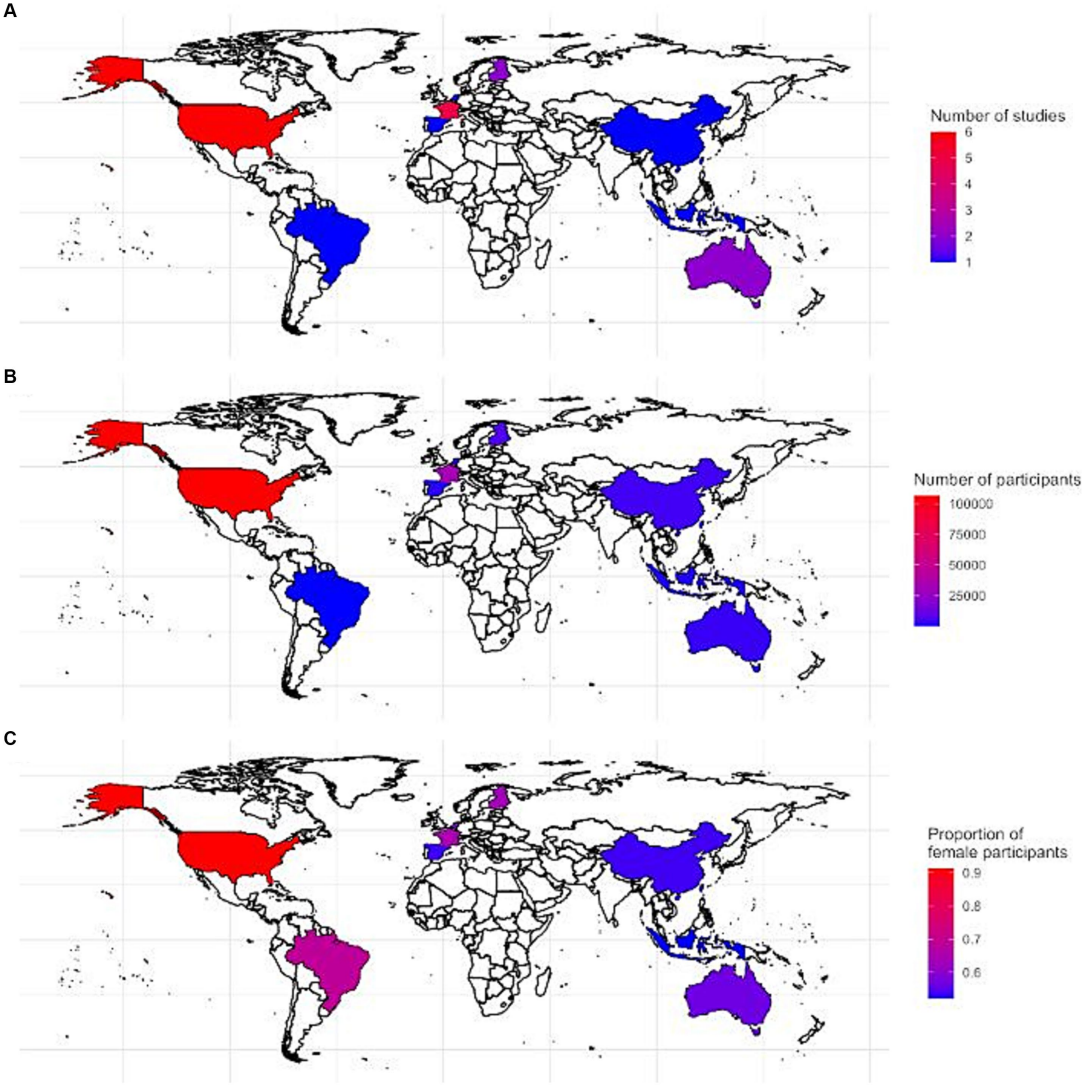
Graphical representation showing the geographical area covered by the studies in the systematic review: **(A)** the number of included studies, **(B)** the total number of participants based on the included studies, and **(C)** the proportion of females in the total amount of included participants per country.

The study characteristics of the included reports are presented in Table 1. The respective sample sizes ranged from 210 (35) to 52,135 (37) participants. Regarding the geographical setting, 10 of the studies were conducted in European countries, namely France (24, 30–32, 38), United Kingdom (28), Finland (19, 29), Spain (33), and the Netherlands (36). Six studies were conducted in the United States (22, 25–27, 34, 37), two studies in Australia (39, 40), one study in Brazil (35), and three studies in Asia, with one study in Taiwan (23), one in Indonesia (21), and one in China (20). Figure 2 provides a graphical representation of the distribution of the geographical areas covered by the studies included in the review. Most studies recruited both male and female participants, with only two studies including exclusively females (26, 37), and one study male participants only (29). The follow-up time ranged from five (24) to 36 (29) years. All but three studies included participants aged 45 years or older at study baseline (22, 29, 31, 37).

**Table 1:** Summary of the baseline characteristics of included studies.

First author, year (Reference)	Study population	Country	Study period	Sample size	Baseline age (years)	Gender (%)	Socio-demographic	Nutrition and lifestyle	Metabolic health	Psychological
Aalto et al. (2023) (19)	Helsinki Aging Study	Finland	4 waves: 1989, 1999, 2009, 2019	*N*_1989_ = 552 *N*_1999_ = 2,396 *N*_2009_ = 1,492 *N*_2019_ = 1,614	≥75 years	Female: 69.0%	X			X
Zhu et al. (2023) (20)	China Health and Retirement Longitudinal Study	China	Baseline: 2013 End of follow-up: 2015	*N* = 4,280	≥60 years	Female: 53.6%	X			X
Oktaviani et al. (2022) (21)	Indonesia Family Life Survey	Indonesia	Baseline: 2007 End of follow-up: 2014	*N* = 1,289	≥60 years	Female: 52.1%	X	X		X
Lee-Bravatti et al. (2021) (22)	Boston Puerto Rican Health Study	United States	Baseline: 2004 End of follow-up: 2009	*N* = 889	≥45 years	Female: 71.4%	X	X	X	X
Lin et al. (2021) (23)	Taiwan Initiatives for Geriatric Epidemiological Research	Taiwan	Baseline: 2011 End of follow-up: 2019	*N* = 467	≥65 years	Female: 54.8%	X	X	X	X
Assmann et al. (2019) (24)	NutriNet-Santé Study	France	Baseline: 2009 End of follow-up: 2016	*N* = 21,407	≥45 years	Female: 72.8%		X		
Cooney and Curl (2019) (25)	Health and Retirement Study (HRS)	United States	Baseline: 1998 End of follow-up: 2012	*N* = 12,108	≥51 years	Female: 52.5%	X	X		
James et al. (2019) (26)	Nurses’ Health Study	United States	Baseline: 2004 End of follow-up: 2012	*N* = 33,326	68 years^*^	All female				X
Kim et al. (2019) (27)	Health and Retirement Study (HRS)	United States	Baseline: 2006 End of follow-up: 2014	*N* = 5,698	>50 years	Female: 61.3%				X
Lassale et al. (2019) (28)	English Longitudinal Study of Aging	United Kingdom	Baseline: 1998 End of follow-up: 2012	*N* = 2,437	≥47 years	Female: 56.5%			X	
Urtamo et al. (2019) (29)	Helsinki Businessmen Study	Finland	Baseline: 1974 End of follow-up: 2010	*N* = 533	≥40 years	All male	X	X	X	
Assmann et al. (2018) (30)	NutriNet-Santé Study	France	Baseline: 1994 End of follow-up: 2007	*N* = 2,249	≥45 years	Female: 46.5%		X		
Assmann et al. (2018) (31)	SU.VI.MAX Study	France	Baseline: 1994 End of follow-up: 2007	*N* = 3,012	≥35 years	Female: 47.6%		X		
Atallah et al. (2018) (32)	SU.VI.MAX Study	France	Baseline: 1994 End of follow-up: 2009	*N* = 2,203	≥45 years	Female: 45.7%		X		
Domènech-Abella et al. (2018) (33)	COURAGE in Europe project	Spain	Baseline: 2011 End of follow-up: 2014	*N* = 1,886	≥50 years	Female: 53.5%	X			
Lai et al. (2018) (34)	Cardiovascular Health Study	United States	Baseline: 1992 End of follow-up: 2015	*N* = 2,622	74.4 years^*^	Female: 63.4%		X		
Rinaldi et al. (2018) (35)	The Porto Alegre Longitudinal Aging Study (PALA)	Brazil	Baseline: 1996 End of follow-up: 2012	*N* = 210	≥60 years	Female: 69.0%	X			X
Jaspers et al. (2017) (36)	The Rotterdam Study	The Netherlands	Baseline: 1990 End of follow-up: 2002	*N* = 3,527	≥55 years	Female: 60.2%	X			
Ma et al. (2017) (37)	Nurses’ Health Study	United States	Baseline: 1980 End of follow-up: 2012	*N* = 52,135	≥33.5 years	All female		X		
Ruhunuhewa et al. (2017) (38)	SU.VI.MAX Study	France	Baseline: 1994 End of follow-up: 2009	*N* = 2,733	≥45 years	Female: 50.6%		X		
Gopinath et al. (2016) (39)	The Blue Mountains Eye Study (BMES)	Australia	Baseline: 1992 End of follow-up: 2004	*N* = 1,609	≥50 years	Female: 56.7%		X		
Gopinath et al. (2016) (40)	The Blue Mountains Eye Study (BMES)	Australia	Baseline: 1992 End of follow-up: 2002	*N* = 1,609	≥50 years	Female: 56.7%		X		

^*^Mean age reported only.

### 3.3 Determinants of successful aging

**Figure 3:**
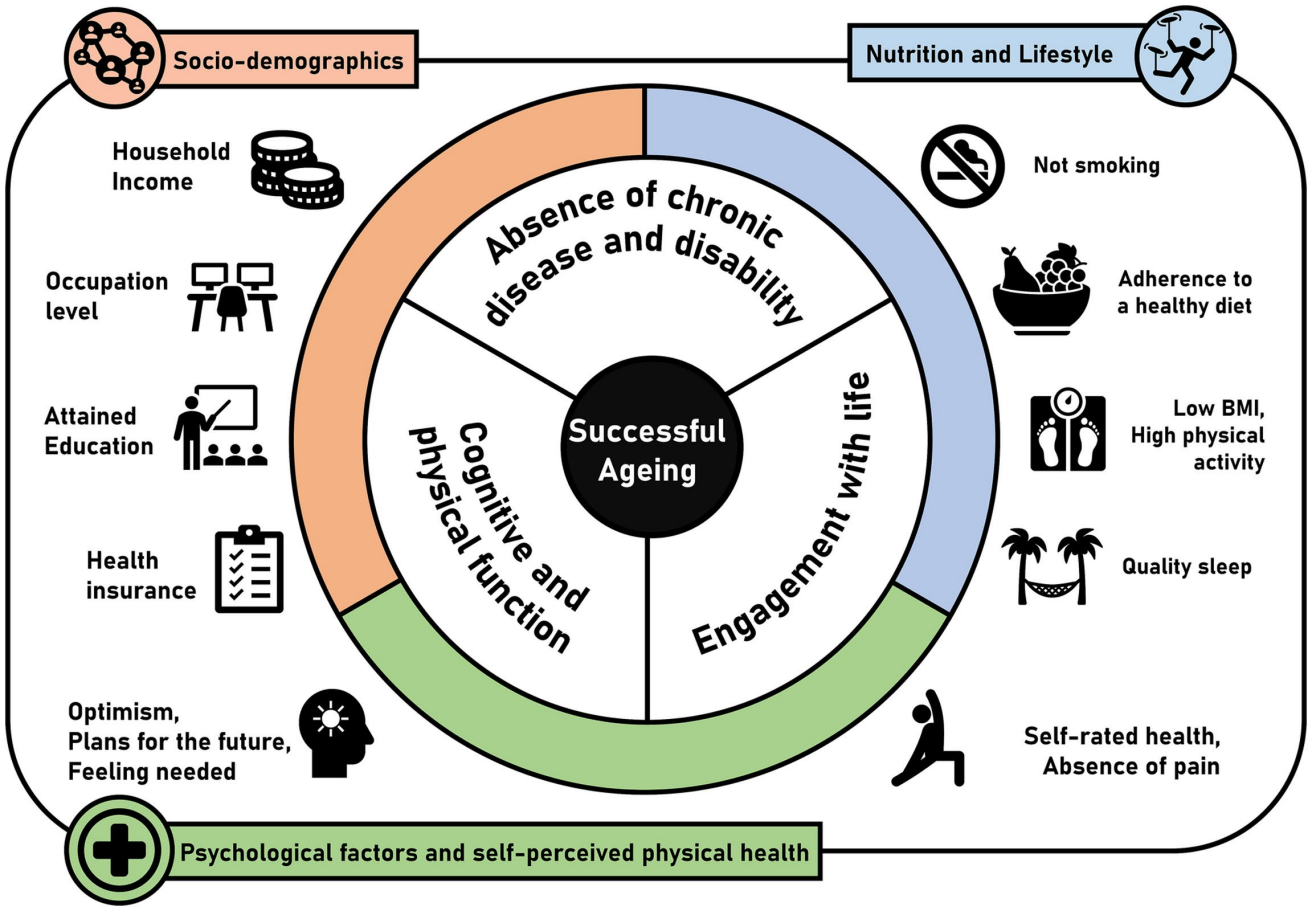
Overview of determinants and indicators of SA from the studies included in the systematic review (*n* = 22).

An overview summarizing the results for the reported associations of socio-demographic, nutritional, lifestyle, psychological, and biological factors with SA is presented in Table 2. Figure 3 additionally provides a summary of the main determinants shown to be statistically significantly associated with SA based on the studies included in the systematic review. More details of these associations are provided below.

**Table 2:** Summary of the main results on the reported determinants of SA.

Domain	Category	Positive	Negative	Null	Mixed
Socio-demographic		↑ Occupation level (33) ↑ Household income (22, 25) ↑ Attained education (22, 25, 33) Health insurance (22)	Black or Hispanic ethnicity (22, 25) Debt (25)	Widowed or separated marital status (33) Rural residence (33) Place of residency in Southern United States (25) Age of oldest living parent (25) Never employed (33) Childhood financial adversity (25)	Female sex (21, 25, 33, 35, 36)
Nutrition and lifestyle	Anthropometrics	↓ BMI (22)	Overweight or obese, or ↑ BMI (25, 29, 38) ↑ Height (37)		
	Lifestyle	Healthy lifestyle^*^ (32) ↑ Physical activity (19, 21, 22, 40) ↑ Quality of sleep (22)	Smoking (21, 22, 25)		
	Dietary patterns	Adherence to a healthy diet^†^ (24, 30, 31, 40)	↑ Energy intake (40)	↑ Glycaemic index and dietary glycaemic load (39)	
	Food groups	↑ Fruit and vegetable intake (40) ↑ Fish intake (40) Medium meat intake (40) ↑ Cereal intake (40) Medium diary intake (40)	↑ Meat intake (40) ↑Biscuit and cake consumption (40) ↑ Sugar consumption (40) ↑ Non-alcoholic drinks (40)	↑ Coffee (29) Moderate alcohol consumption (29)	
	Macronutrients	↑ Fiber intake (39) ↑ n3-PUFAs intake (34) ↑ EPA levels (34) ↑ DPA levels (34)		↑ Carbohydrate intake (39) ↑ ALA levels (34) ↑ DHA levels (34)	
Metabolic Health	Biological markers		↑ CRP level trajectories^‡^ (28) ↑ Total cholesterol (29) ↓ Metabolic health§ (23, 38) Metabolic syndrome (23)	↑ Triglyceride levels (29) ↑ Systolic and diastolic blood pressure (29)	
Self-perceived health		↑ Self-rated health (19, 22, 29) ↑ Absence of pain that limits function (20, 24, 30–32, 36, 38)	↓ Childhood health (25)	↑ Self-rated physical fitness (29)	
Psychological		↑ Optimism (26, 27) ↑ Plans for the future (19) ↑ Feeling needed (19)			

^*^Healthy lifestyle defined as: healthy weight, former or never smoker, moderate to high physical activity, alcohol consumption ≤ 12 g/d for women or ≤ 24 g/d for men, and high dietary quality (32). ^†^Healthy diet entails: adherence to guidelines of the French Nutrition and Health Program, the Literature-based Adherence Score to the Mediterranean Diet, and a modified version of the Australian diet quality index, based on the Dietary Guidelines for Australian Adults and the Australian Guide to Healthy Eating (24, 30, 31, 40). ^‡^The four CRP trajectories identified were as follows: “stable-low” (baseline mean CRP of 1.33 mg/L, and remaining < 3 mg/L over follow-up); “medium-to-high” (baseline mean CRP of 2.7 mg/L rising to 5.3 mg/L over follow-up); “high-to-medium” (baseline mean CRP of 6.6 mg/L, and decreasing to 2.4 mg/L over follow-up); and “stable-high” (CRP levels from 5.7 to 7.5 over the study duration) (28). §Impaired metabolic health present when corresponding to two or more of the following criteria: 1. triglycerides ≤ 1.7 mmol/L or lipid-lowering drugs; 2. systolic blood pressure ≥ 130 mm Hg, diastolic blood pressure ≥ 85 mm Hg, or use of antihypertensive drugs; 3. glucose ≥ 5.6 mmol/L or use of antidiabetic medication; 4. high-density lipoprotein cholesterol < 1.03 mmol/L for men and < 1.29 mmol/L for women (38). The arrows demonstrate whether the exposure levels associated with successful aging are low (downwards arrow, ↓) or high (upwards arrow, ↑). ALA, amino leavulinic acid; BMI, body mass index; CRP, c-reactive protein; DHA, docosahexaenoic acid; DPA, docosapentaenoic acid; EPA, eicosapentaenoic acid; N3-PUFA, omega 3 polyunsaturated fatty acids.

#### 3.3.1 Socio-demographic factors

Among the demographic factors, sex was the most reported determinant of SA (n_studies_ = 5, n_participants_ = 19,020). Female sex was associated with lower odds for SA in three studies (*n* = 13,607) (21, 25, 36). Vice versa, it was associated with higher odds for SA in another study (*n* = 3,527) (35). One study found no association between sex and SA (*n* = 1,886) (33). Being of black or Hispanic ethnicity, as well as being in debt were negatively associated with SA in the Health and Retirement Study from the United States (*n* = 12,108) (25). Furthermore, in the same population, a higher household income was positively associated with SA, but not childhood financial adversity (25). In the Spanish population of the COURAGE Europe Project, a higher occupational status was positively associated with SA, whereas no association was present in individuals that never were employed (33).

Results about the association between residency and SA have been reported in two studies. Cooney et al. (25) did not find an association between SA and residency in the south compared to other regions in the United States. The South region was described “to have the lowest median household income, and highest poverty rate and rate of uninsured individuals in the population.” In a Spanish study, rural residence as compared to urban residence was associated with higher odds for SA (33). This entails several dimensions such as demographics, access to health services, intergenerational and other social relationships (41). Furthermore, no association with SA was found for marital status (33) and age of the oldest living parent indicating family longevity (25).

#### 3.3.2 Nutrition and lifestyle factors

Anthropometrics and lifestyle factors were the most explored factors in the domain *nutrition and lifestyle*. Four studies reported results on the role of anthropometric indicators of nutritional status as determinants of SA suggesting that taller height as an accumulation of “genetic endowment and various early-life exposures” (37) and higher body mass index (25, 29, 38) were negatively associated with SA. Interestingly, Ma et al. (37) reported that the lower odds for SA in taller individuals could be modulated by a diet rich in fruits and vegetables. Four studies reported positive associations between adherence to dietary recommendations, including the French dietary guidelines (24, 30), the Australian dietary recommendations (40), and the Mediterranean diet (31) with SA. Concerning beverages, low alcohol consumption was associated with SA when examined in context of an overall healthy diet (31, 32) but not when the univariate association was examined (31). However, moderate alcohol consumption was associated with SA in another study by Urtamo et al. (29). The same study also reported null findings on coffee consumption. Gopinath et al. (40) reported null findings on the consumption of non-alcoholic beverages and the association with SA. In addition to dietary patterns, several studies further explored the association between specific nutrients and SA. Gopinath et al. (39) reported that high fiber intake was positively, while a high glycaemic index was negatively associated with SA. Lai et al. (34) found that higher levels of circulating omega 3 fatty acids were positively associated with SA. More specifically, high eicosa- and docosapentaenoic acid were positively associated, whereas alpha linolic and docosahexaenoic acid levels were not associated with SA, respectively (34).

Beyond individual behaviors, several studies explored combination of lifestyle factors in relation to SA. Based on data from the French” Supplémentation en Vitamines et Minéraux Antioxydants” (SU.VI.MAX) cohort, Atallah et al. (32) assessed the combined impact of lifestyle factors (weight, smoking status, physical activity, alcohol consumption, and diet) modeled as a healthy lifestyle index on SA. Each point increase in the lifestyle index was associated with an 11% higher probability of SA. The proportions of SA attributable to specific factors were 7.6, 6.0, 7.8, and 16.5% for body mass index, physical activity, diet quality, and smoking status, respectively. Lee-Bravatti et al. (22) explored a combination of lifestyle factors that included physical activity, smoking, sleep, and diet among older Puerto Rican adults. The authors found that the constructed lifestyle score was more strongly associated with SA than individual behaviors alone, suggesting that there are synergistic effects at play. Furthermore, the study highlighted physical activity, never smoking, and higher quality of sleep to play a dominant role in affecting SA. Overall, these studies underline the importance of healthy lifestyle habits at midlife as determinants of overall health during aging.

#### 3.3.3 Biological factors

In recent years, the number of studies that explored biological pathways as intermediary determinants of SA has increased. Ruhunuhewa et al. (38) reported that impaired metabolic health, defined by high triglyceride levels or lipid-lowering drug use, high blood pressure, high blood glucose levels, high total cholesterol, and low high-density lipoprotein cholesterol, was negatively associated with SA. Furthermore, Lassale et al. (28) found that in individuals aged between 47 and 87 years at baseline, increasing CRP levels over the course of 10 years of follow-up presented lower odds for SA than those that had stable low CRP trajectories. In a more recent study, Lin et al. (23) explored the association of the metabolic syndrome and its components (central obesity, elevated blood glucose, low high-density lipoprotein cholesterol, and hypertension) with SA. The results supported the previously reported link between metabolic health and SA, showing that it may be important not only for physical health, but also may determine psychological well-being.

#### 3.3.4 Psychological factors and well-being

The association of psychological factors and well-being with SA has been explored by four studies. In the Health and Retirement Study, higher optimism was associated with SA for both males (27) and females (26, 27), although the association was found to be stronger in males. Self-perceived health was considered as an indicator for SA in two studies. Urtamo et al. (29) assessed self-rated health and found a positive association with SA. Cooney and Curl (25) investigated self-reported childhood health and reported poor health to be negatively associated with SA. Furthermore, based on data from the Helsinki Aging Study, Aalto et al. observed that participants who characterized themselves as “feeling needed” and “having plans for the future” had higher odds for SA (19). Most studies assessed SA as a binary outcome, except from six studies that used a continuous outcome, i.e., by means of a numerical score (22, 27, 28, 32, 33, 36). Following the conceptual definition of Rowe and Kahn’s model (4), all selected studies included in our review defined SA as a multidimensional outcome and included different aspects of the major proposed domains. Specific components of SA that depicted the first two domains, namely *absence of chronic disease* as well as *disability and good physical and cognitive functioning* included favorable state of respiratory function (28, 39, 40), blood pressure (23, 28), cardiovascular health (39, 40), absence of pain that limits function (20, 24, 30–32, 36, 38), and self-rated health (19, 24, 30–32, 36, 38). The *engagement with life* domain was largely defined as the lack of limitations on social functioning in six out of nine studies (24, 25, 30–32, 36, 38). One study included *happiness* as an additional component of SA (29). Methods of assessment and measurement scales of the individual components of SA varied extensively throughout the studies with no standardized definition of SA used across the studies.

### 3.4 Risk of bias assessment

**Figure 4:**
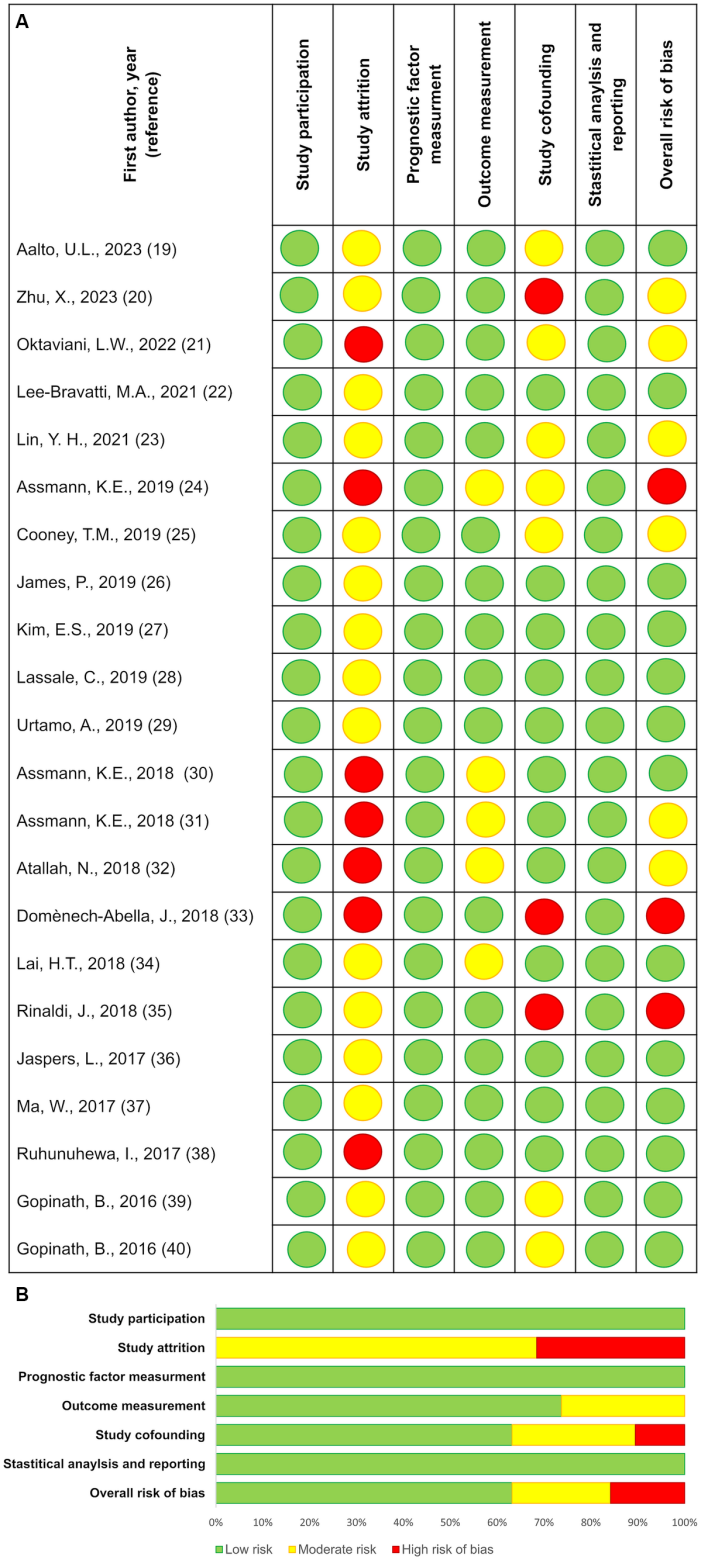
Results from the risk of bias assessments according to the Quality in Prognosis Studies (QUIPS) tool, showing ratings for the separate risk of bias domains, as well as the overall study bias. Domain 1: Study participation: to judge the risk of selection bias; Domain 2: Study attrition: to judge the risk of attrition bias; Domain 3: Prognostic factor measurement: to judge the risk of measurement bias in the measurement of the prognostic factor(s); Domain 4: Outcome measurement: to judge the risk of measurement bias in the measurement of the outcome; Domain 5: Study confounding: to judge the risk of bias due to confounding; Domain 6: Statistical analysis and reporting: to judge the risk of bias due to statistical analysis and result reporting. **(A)** Risk of bias for individual studies; **(B)** Summary risk of bias for each domain.

The results of the RoB assessment are presented in Figure 4. Overall, the RoB was *low* in 13 out of 22 included studies, while six and three studies presented *moderate* and *high* RoB, respectively. The high RoB score was explained by ratings on study attrition and lack of confounder control. Moreover, study attrition was rated *medium* RoB in 15 out of 22 studies, and as *high* in the remaining seven studies.

## 4 Discussion

The current review provides a summary of the recent research on determinants of SA, including socio-demographic factors, nutrition, lifestyle, biological pathways, psychological health, and well-being. Overall, a higher socio-economic status, adherence to healthy plant-based diet such as the Mediterranean diet, not smoking, being physically active, and being metabolically healthy, were the factors associated with higher odds for SA. Newly emerging aspects of increasing importance in research on SA include psychological factors and well-being.

The review confirms past research highlighting the value of a healthy lifestyle throughout the life course as a major determinant of SA (32). It essentially points to that adopting an overall healthy lifestyle rather than focusing on avoidance of risk factors seems to be the key to long-term sustained health. These findings largely correspond to the results by previous reviews (8, 15, 42). Among individual determinants, adherence to healthy dietary patterns, such as the Mediterranean diet, have been pronouncedly associated with higher chances for SA (24, 30, 31, 40). In contrast, individual dietary components, such as high carbohydrate intake and high glycaemic index of a diet were not associated with SA (39). The current review found moderation in the consumption of alcohol to be positively associated with SA, albeit in the context of following an overall healthy diet. In contrast, in previous review, Kralj et al. (15) reported mixed findings for the same association. Potential explanations for these discrepant findings could be sought in the assessment and categorization of drinking patterns among studies, the type of alcohol consumed, and the different reference categories used. Our results are in line with results from a more recent systematic review that reported that as compared to complete abstinence moderate alcohol consumption was positively associated with healthy aging (10). Furthermore, our results revealed that sleeping habits including sleeping time and quality were strongly associated with SA (22). This evidence comes in support of previous research showing that sleep is associated with improved cognitive functioning and may be protective against aging-related cognitive decline (43).

Another highlight of the current review is the role of the metabolic health as an important biological intermediate factor for SA (38). Suboptimal levels of lipid metabolism biomarkers, high blood pressure, and high glucose levels over prolonged time periods have been long known to predispose higher risk of age-related chronic diseases, including cardiovascular disease (44) and type 2 diabetes (44), as well as overall mortality, and have also been negatively associated with SA (28). Metabolic disorders are associated with chronic systemic inflammation leading to the generation of reactive oxygen species and oxidative stress that exacerbates the aging process, functional decline, and disease development (45–47). Additional aging phenotypes include the declining functions of the immune system, called immunosenescence, the senescence-associated secretory phenotype, shortened telomeres and decreased telomerase activity. All of these may play an important role as biological determinants for successful aging and further research would be warranted to explore relevant biomarkers associated with these processes individually and conjointly.

Our review further identified an increasing number of studies reporting on psychological factors and well-being in relation to SA. One of these factors reportedly associated with SA was optimism (26, 27). Previous research reported on the inverse association between optimism and chronic disease outcomes, incl. Cardiovascular disease, cancer, mortality, and cognitive decline (48–50). Positive life orientation may affect the adoption and maintenance of behaviors physical activity, less smoking and adherence to a healthy diet (51), which are known risk factors for cardiovascular disease. It is also plausible that optimism and pessimism affect health through biological mechanisms independent of health behaviors. Optimism has been associated with an improved immune response (52), higher levels of antioxidants in the blood (53), and advantageous high density lipoprotein cholesterol levels (54). If confirmed by future research these findings would suggest that the cultivation of optimism may be an important feature of designing strategies promoting SA. In addition, various aspects related to the socio-economic status, such as ethnicity (22, 25), marital status (33), place of residence (25, 33) and debt (25) were also found as determinants of SA. Taken together, our findings go beyond evidence provided by previous reviews, i.e., Kralj et al. (15) by extending the list of socio-economic and cognitive psycho-social factors as determinants of SA. The role of early life health assessment has been also among the newly identified factors reflecting the increased importance in research of understanding life-course determinants of health. This new evidence further underlines the notion that SA, apart from biological and lifestyle aspects, can be strongly influenced by early childhood experiences, mental health and well-being along with social justice and financial inequity.

The evidence of this review originates from well-conducted longitudinal studies, most of which were attributed a low RoB. However, the summary of results is challenging due to several methodological limitations. These include (a) the lack of a standardized definition of successful aging, (b) the large variation of methods employed for the operationalization of its multiple components, and (c) the under-representation of various population groups, particularly from less developed regions and lower socio-economic status. The results from the review should therefore be interpreted considering the differences in both measurement and definition of SA as the outcome of interest. So far, there has not been an accepted universal definition for SA and various operational definitions have been used in research (55). Next to the Rowe and Kahn’s model (4), psychological models of SA have been proposed, such as the concept proposed by Baltes and Baltes (56), which puts more emphasis on the adaptation to the older stages of life and limitations to functional capacity. The model of “Aging well” by Fernandez-Ballesteros et al. (55) further includes *activities of daily living*, *physical and cognitive functioning*, *social participation and engagement*, and *positive affect and control* as additional domains within the definition of SA. Other researchers such as Young et al. (57, 58) attempted to depict further multidimensional aspects in the definition allowing assessment of SA on a continuous rather than dichotomous scale.

One of the main challenges in conceptualizing SA is combining objective criteria (e.g., functional abilities and the number of chronic conditions) and subjective criteria (e.g., satisfaction with life). To combine information about objective and subjective criteria, Kok et al. (59) quantified SA according to the number of indicators in which individuals showed successful trajectories over the life course. Cosco et al. (60) created the *a priori* continuous Successful Aging Index using items identified by systematic reviews of operational definitions and lay perspectives of SA which included seven items: *maintenance of interest, absence of loneliness, optimism, self-rated health, cognitive functioning,* and *instrumental activity of daily living*. Using confirmatory factor analysis, Kleineidam et al. (61) compared five methods for assessing objective and subjective successful aging (physical health, cognitive health, disability, well-being, and social engagement) and demonstrated that these domains can be combined into a multidimensional construct. Overall, the studies included in the current review predominantly defined SA based on objective measurements of health and functionality and were less focused on a wider range of subjective criteria such as the individuals’ perceptions of their own health and well-being. Recent studies showed that life satisfaction (7), purpose in life (7, 62, 63), and social engagement (14) contributed to SA and therefore should be included in operationalizing SA. Overall, more work is needed on developing a well-constructed definition for SA that balances subjective and objective aspects based on measurements of physiological health, well-being, and social engagement. Future research is warranted to provide agreed-upon operationalization of SA and standardized measurements of its separate components, to facilitate comparable findings and an adequate summary of main tendencies in research.

The strengths of the current systematic review include its actuality based on inclusion of studies published in the past 7 years, thus providing an overview of recent trends and novel findings in research on determinants and indicators of SA. Compared to previous reviews that focused on single factors, this review included a wide range of factors explored as determinants and indicators of SA. The definition of SA was carefully considered and only studies that adhered to the multidimensional concept of SA were included to allow for a better overview on the evolution of its definition and the detection of methodological gaps. The current review was conducted according to the most recent guidelines for conducting and publishing systematic reviews and included a careful assessment of study quality using predefined criteria. Furthermore, only longitudinal studies were included, which allows inferences on temporality of found associations. However, with the observational nature of the studies, causal assumptions cannot be fully supported. Most included studies proved to be of high quality, although some studies were limited by the lack of a proper evaluation of study attrition and insufficient control for potential confounding. Concerning the study attrition, it remains important to report to conduct descriptive analysis on participants that were lost to follow-up, whether they differed from the participants that completed the study, and how the study findings may thus be biased. Future studies should consider methodological limitations and provide sufficient detail on the planning and statistical analysis. The geographical coverage of the current study was also limited, with majority of studies representing populations from Europe, the United States, and Australia. Therefore, current findings may not be generalizable to populations living outside of the global north. Furthermore, the majority of studies were based on older populations which restricted the possibility to explore possible differences in studied exposures according to age group. Future studies including wider age ranges of participants, i.e., representing younger age groups, are warranted to allow a more detailed evaluation of the time and duration of exposure.

Finally, the current review aimed to depict a multidimensional definition of SA, thereby excluding studies that evaluated other age-related outcomes such as disease-free aging or healthy life-expectancy. Despite such outcomes being explored on a wider basis in epidemiology and public health research, they may not sufficiently cover additionally important aspects of the aging concept beyond absence of diseases and disability. Our work and that of previous reviews on SA as a multidimensional outcome underlines the importance of employing a holistic view to define the quality of life at older ages. Understanding the complexity of SA and subsequently fine-tuning novel approaches to address it may hold the key to adding life to years, beyond years to life.

In conclusion, this systematic review provides a summary of the recent evidence on the determinants of successful aging and highlights the importance of healthy living, monitoring metabolic risk along with sustaining mental health and well-being in adult life. Emerging research highlights the role of psycho-social factors and early life health as determinants of SA. Future research is warranted to develop a standardized definition of SA and explore the synergistic effects of its components. Investment in further methodological and research work on SA would pave the way toward development of adequate health promotion policies in aging societies.

## Data availability statement

The original contributions presented in the study are included in the article/Supplementary material, further inquiries can be directed to the corresponding author.

## Author contributions

CR: Investigation, Writing – original draft, Writing – review & editing. CG: Visualization, Writing – review & editing. RA: Investigation, Writing – review & editing. MB: Writing – review & editing. KA: Conceptualization, Funding acquisition, Investigation, Methodology, Project administration, Writing – original draft, Writing – review & editing.
