# Supplementary material for: Determinants and indicators of successful aging as a multidimensional outcome: a systematic review of longitudinal studies
Source: Front Public Health. 2023 Nov 21;11:1258280. doi: 10.3389/fpubh.2023.1258280 (PMC10703300; doi:10.3389/fpubh.2023.1258280)
Supplement: Supplementary file 1 [file Table_1.DOCX]

**Supplemental Tables**

**STable 1 Summary of inclusion and exclusion criteria for the current systematic review**

| **Inclusion and exclusion criteria** | **Rationale for the criteria** |
| --- | --- |
| **Inclusion criteria** |  |
| Published between 08-2016 and 06-2023 | To include research not previously included in the systematic reviews on determinants of successful ageing |
| Articles full text published in English | To ensure correct interpretation of study findings |
| Original research articles published in peer reviewed journals | To ensure a good quality standard for included studies |
| Longitudinal observational design | To increase the evidence of an association being causal to the successful ageing outcome |
| Primary aim includes characterization of determinants of successful ageing | To fit the goal of the current systematic review, gathering recent evidence and trends on determinants of successful ageing |
| A multidimensional definition of successful ageing based on ≥3 domains of Rowe & Kahn | To ensure a baseline agreement on successful ageing definition for comparison of findings |
| Community-dwelling populations | At baseline institutionalized populations would conflict with the definition of successful ageing by Rowe & Kahn |
| Populations aged >18 years at baseline | Younger populations will not allow for longitudinal assessment of determinants successful ageing in older age |
| **Exclusion criteria** |  |
| Secondary source articles and methodological studies | Studies with no original quantitative data will be excluded to avoid including duplicate data |
| Animal/experimental laboratory studies | Animal and experimental studies are not within the scope of this review |
| SA definitions not including domains of SA as defined by Rowe & Kahn as a composite measure | To ensure a baseline agreement on successful ageing definition for comparison of findings and inclusion of studies assessing determinants of successful ageing as a holistic, multidimensional concept |
| Studies in special groups of the population | To provide an overview of determinants of successful ageing in the general population |

**STable 2 Risk of bias assessment results domain 1: Study participation**

| **First author, year (*Ref*)** | 1. **Adequate participation in the study by eligible persons** | 1. **Description of the source population or population of interest** | 1. **Description of the baseline**   **study sample** | 1. **Adequate description of the sampling frame and**   **recruitment** | 1. **Adequate description of the period and place of**   **recruitment** | 1. **Adequate description of inclusion and exclusion criteria** |
| --- | --- | --- | --- | --- | --- | --- |
| Aalto, U.L., 2023 (1) | Low bias | Low bias | Low bias | Low bias | Low bias | Low bias |
| Zhu, X., 2023 (2) | Low bias | Low bias | Low bias | Low bias | Low bias | Low bias |
| Oktaviani, L.W., 2022 (3) | Low bias | Low bias | Low bias | Low bias | Low bias | Low bias |
| Lee-Bravatti, M. A., 2021 (4) | Low bias | Low bias | Low bias | Low bias | Low bias | Low bias |
| Lin, Y. H., 2021 (5) | Low bias | Low bias | Low bias | Low bias | Low bias | Low bias |
| Assmann, K.E., 2019 (6) | Moderate bias | Low bias | Low bias | Low bias | Low bias | Low bias |
| Cooney, T.M., 2019 (7) | Low bias | Low bias | Low bias | Low bias | Low bias | Moderate bias |
| James, P., 2019 (8) | Moderate bias | Low bias | Low bias | Low bias | Low bias | Low bias |
| Kim, E.S., 2019 (9) | Low bias | Low bias | Low bias | Low bias | Low bias | Low bias |
| Lassale, C., 2019 (10) | Moderate bias | Low bias | Low bias | Low bias | Low bias | Low bias |
| Urtamo, A., 2019 (11) | Low bias | Low bias | Low bias | Low bias | Low bias | Low bias |
| Assmann, K.E., 2018 (12) | Moderate bias | Low bias | Low bias | Low bias | Low bias | Low bias |
| Assmann, K.E., 2018 (13) | Moderate bias | Low bias | Low bias | Low bias | Low bias | Low bias |
| Atallah, N., 2018 (14) | Moderate bias | Low bias | Low bias | Low bias | Low bias | Low bias |
| Domènech-Abella, J., 2018 (15) | Moderate bias | Low bias | Moderate bias | Low bias | Low bias | Low bias |
| Lai, H.T., 2018 (16) | Low bias | Low bias | Low bias | Low bias | Low bias | Low bias |
| Rinaldi, J., 2018 (17) | Moderate bias | Low bias | Low bias | Low bias | Low bias | Moderate bias |
| Jaspers, L., 2017 (18) | Moderate bias | Low bias | Low bias | moderate bias | Low bias | Low bias |
| Ma, W., 2017 (19) | Moderate bias | Low bias | Low bias | Low bias | Low bias | Low bias |
| Ruhunuhewa, I., 2017 (20) | Moderate bias | Low bias | Low bias | Low bias | Low bias | Low bias |
| Gopinath, B., 2016 (21) | Low bias | Low bias | Moderate bias | Low bias | Low bias | Low bias |
| Gopinath, B., 2016 (22) | Low bias | Low bias | Low bias | Low bias | Low bias | Low bias |

**STable 3 Risk of bias assessment results domain 2: Study attrition**

| **First author, year (*Ref*)** | 1. **Adequate response rate for study participants** | 1. **Description of attempts to collect information on participants who dropped out** | 1. **Reasons for loss to follow-up are provided** | 1. **Adequate description of participants lost to follow-up** | 1. **There are no important differences between participants who completed the study and those who did not** |
| --- | --- | --- | --- | --- | --- |
| Aalto, U.L., 2023 (1) | Moderate bias | Moderate bias | Low bias | Moderate bias | Low bias |
| Zhu, X., 2023 (2) | Moderate bias | Moderate bias | Moderate bias | Moderate bias | Moderate bias |
| Oktaviani, L.W., 2022 (3) | Moderate bias | Moderate bias | Moderate bias | High bias | High bias |
| Lee-Bravatti, M. A., 2021 (4) | High bias | Moderate bias | Moderate bias | Moderate bias | Moderate bias |
| Lin, Y. H., 2021 (5) | Low bias | Moderate bias | Moderate bias | Moderate bias | Moderate bias |
| Assmann, K.E., 2019 (6) | High bias | High bias | Moderate bias | High bias | High bias |
| Cooney, T.M., 2019 (7) | Low bias | Low bias | Moderate bias | Moderate bias | Moderate bias |
| James, P., 2019 (8) | Low bias | Moderate bias | Moderate bias | Moderate bias | Moderate bias |
| Kim, E.S., 2019 (9) | Low bias | Moderate bias | Moderate bias | Moderate bias | Moderate bias |
| Lassale, C., 2019 (10) | High bias | Moderate bias | Low bias | Moderate bias | Moderate bias |
| Urtamo, A., 2019 (11) | Low bias | High bias | Low bias | Low bias | low bias |
| Assmann, K.E., 2018 (12) | Moderate bias | High bias | Low bias | High bias | High bias |
| Assmann, K.E., 2018 (13) | Moderate bias | High bias | Low bias | High bias | High bias |
| Atallah, N., 2018 (14) | High bias | High bias | Moderate bias | High bias | High bias |
| Domènech-Abella, J., 2018 (15) | Moderate bias | High bias | Moderate bias | High bias | High bias |
| Lai, H.T., 2018 (16) | Low bias | Moderate bias | Moderate bias | Moderate bias | Moderate bias |
| Rinaldi, J., 2018 (17) | Moderate bias | Low bias | Moderate bias | Low bias | Moderate bias |
| Jaspers, L., 2017 (18) | Moderate bias | Moderate bias | Moderate bias | High bias | High bias |
| Ma, W., 2017 (19) | Low bias | Moderate bias | Moderate bias | Moderate bias | Moderate bias |
| Ruhunuhewa, I., 2017 (20) | High bias | High bias | High bias | Low bias | Moderate bias |
| Gopinath, B., 2016 (21) | Moderate bias | Moderate bias | Low bias | Low bias | Low bias |
| Gopinath, B., 2016 (22) | Moderate bias | Moderate bias | Low bias | Moderate bias | Moderate bias |

**Table 4 Risk of bias assessment results domain 3: Prognostic factor measurement**

| **First author, year (*Ref*)** | 1. **A clear definition or**   **description of the PF is provided** | 1. **Method of PF**   **measurement is adequately valid and**  **reliable** | 1. **Continuous variables**   **are reported or appropriate cut points are used** | 1. **The method and setting of**   **measurement of PF is the same for all study participants** | 1. **Adequate proportion**   **of the study sample has complete data for the PF** | 1. **Appropriate methods of imputation are used for missing PF data** |
| --- | --- | --- | --- | --- | --- | --- |
| Aalto, U.L., 2023 (1) | Low bias | Low bias | Low bias | Low bias | Low bias | Low bias |
| Zhu, X., 2023 (2) | Low bias | Low bias | Low bias | Low bias | Low bias | Low bias |
| Oktaviani, L.W., 2022 (3) | Low bias | Low bias | Low bias | Low bias | Low bias | Low bias |
| Lee-Bravatti, M. A., 2021 (4) | Low bias | Low bias | Low bias | Low bias | Low bias | Low bias |
| Lin, Y. H., 2021 (5) | Low bias | Low bias | Low bias | Low bias | Low bias | Low bias |
| Assmann, K.E., 2019 (6) | Low bias | Low bias | Low bias | Low bias | Moderate bias | Low bias |
| Cooney, T.M., 2019 (7) | Low bias | Moderate bias | Low bias | Low bias | Low bias | Low bias |
| James, P., 2019 (8) | Moderate bias | Low bias | Low bias | Low bias | Moderate bias | Low bias |
| Kim, E.S., 2019 (9) | Low bias | Low bias | Low bias | Low bias | Low bias | Low bias |
| Lassale, C., 2019 (10) | Low bias | Low bias | Low bias | Low bias | Moderate bias | Low bias |
| Urtamo, A., 2019 (11) | Low bias | Moderate bias | Low bias | Low bias | Moderate bias | Low bias |
| Assmann, K.E., 2018 (12) | Low bias | Low bias | Low bias | Low bias | Moderate bias | Low bias |
| Assmann, K.E., 2018 (13) | Low bias | Low bias | Low bias | Low bias | Moderate bias | Low bias |
| Atallah, N., 2018 (14) | Moderate bias | Moderate bias | Low bias | Low bias | High bias | Low bias |
| Domènech-Abella, J., 2018 (15) | Low bias | Low bias | Low bias | Low bias | Low bias | Low bias |
| Lai, H.T., 2018 (16) | Low bias | Low bias | Low bias | Low bias | Moderate bias | Moderate bias |
| Rinaldi, J., 2018 (17) | Low bias | Low bias | Low bias | Low bias | Low bias | Low bias |
| Jaspers, L., 2017 (18) | Low bias | Low bias | Low bias | Low bias | Low bias | Low bias |
| Ma, W., 2017 (19) | Low bias | Moderate bias | Low bias | Low bias | Low bias | Low bias |
| Ruhunuhewa, I., 2017 (20) | Low bias | Low bias | Low bias | Low bias | Moderate bias | Low bias |
| Gopinath, B., 2016 (21) | Low bias | Low bias | Low bias | Low bias | Moderate bias | Low bias |
| Gopinath, B., 2016 (22) | Low bias | Low bias | Low bias | Low bias | Moderate bias | Low bias |

**STable 5 Risk of bias assessment results domain 4: Outcome measurement**

| **First author, year (*Ref*)** | 1. **A clear definition of the outcome is provided** | 1. **Method of outcome measurement used is adequately valid and reliable** | 1. **The method and setting of outcome measurement is the same for all study participants** |
| --- | --- | --- | --- |
| Aalto, U.L., 2023 (1) | Low bias | Low bias | Low bias |
| Zhu, X., 2023 (2) | Low bias | Low bias | Low bias |
| Oktaviani, L.W., 2022 (3) | Low bias | Moderate bias | Low bias |
| Lee-Bravatti, M. A., 2021 (4) | Low bias | Low bias | Low bias |
| Lin, Y. H., 2021 (5) | Low bias | Low bias | Low bias |
| Assmann, K.E., 2019 (6) | Low bias | Moderate bias | Low bias |
| Cooney, T.M., 2019 (7) | Low bias | Moderate bias | Low bias |
| James, P., 2019 (8) | Low bias | Low bias | Low bias |
| Kim, E.S., 2019 (9) | Low bias | Low bias | Low bias |
| Lassale, C., 2019 (10) | Moderate bias | Low bias | Low bias |
| Urtamo, A., 2019 (11) | Low bias | Moderate bias | Low bias |
| Assmann, K.E., 2018 (12) | Low bias | Moderate bias | Low bias |
| Assmann, K.E., 2018 (13) | Low bias | Low bias | Low bias |
| Atallah, N., 2018 (14) | Low bias | Moderate bias | Low bias |
| Domènech-Abella, J., 2018 (15) | Low bias | Moderate bias | Low bias |
| Lai, H.T., 2018 (16) | Low bias | Moderate bias | Low bias |
| Rinaldi, J., 2018 (17) | Moderate bias | Moderate bias | Low bias |
| Jaspers, L., 2017 (18) | Low bias | Low bias | Low bias |
| Ma, W., 2017 (19) | Low bias | Low bias | Low bias |
| Ruhunuhewa, I., 2017 (20) | Low bias | Low bias | Low bias |
| Gopinath, B., 2016 (21) | Low bias | Moderate bias | Low bias |
| Gopinath, B., 2016 (22) | Low bias | Moderate bias | Low bias |

**STable 6 Risk of bias assessment results domain 5: Study confounding**

| **First author, year (*Ref*)** | **All important confounders are measured** | **Clear definitions of the important confounders measured are provided** | **Measurement of all important confounders is adequately valid and reliable** | **The method and setting of confounding measurement are the same for all study participants** | **Appropriate methods are used if imputation is used for missing confounder data** | **Important potential confounders are accounted for in the analysis** |
| --- | --- | --- | --- | --- | --- | --- |
| Aalto, U.L., 2023 (1) | Moderate bias | Low bias | Moderate bias | Low bias | Low bias | Low bias |
| Zhu, X., 2023 (2) | Moderate bias | Low bias | Moderate bias | Moderate bias | Moderate bias | Low bias |
| Oktaviani, L.W., 2022 (3) | Moderate bias | Moderate bias | Moderate bias | Low bias | Low bias | Low bias |
| Lee-Bravatti, M. A., 2021 (4) | Low bias | Low bias | Moderate bias | Low bias | Low bias | Low bias |
| Lin, Y. H., 2021 (5) | Moderate bias | Low bias | Moderate bias | Moderate bias | Low bias | Moderate bias |
| Assmann, K.E., 2019 (6) | Low bias | Low bias | Moderate bias | Low bias | Low bias | Moderate bias |
| Cooney, T.M., 2019 (7) | Low bias | Low bias | Moderate bias | Low bias | Low bias | Moderate bias |
| James, P., 2019 (8) | Low bias | Moderate bias | Moderate bias | Low bias | Low bias | Low bias |
| Kim, E.S., 2019 (9) | Low bias | Low bias | Low bias | Low bias | Low bias | Low bias |
| Lassale, C., 2019 (10) | Low bias | Low bias | Low bias | Low bias | Low bias | Low bias |
| Urtamo, A., 2019 (11) | Moderate bias | Low bias | Low bias | Low bias | Low bias | Low bias |
| Assmann, K.E., 2018 (12) | Low bias | Low bias | Low bias | Low bias | Low bias | Low bias |
| Assmann, K.E., 2018 (13) | Low bias | Low bias | Low bias | Low bias | Low bias | Moderate bias |
| Atallah, N., 2018 (14) | Low bias | Low bias | Low bias | Low bias | Low bias | Low bias |
| Domènech-Abella, J., 2018 (15) | High bias | Low bias | Low bias | Low bias | Low bias | High bias |
| Lai, H.T., 2018 (16) | Low bias | Low bias | Low bias | Low bias | Low bias | Low bias |
| Rinaldi, J., 2018 (17) | Moderate bias | Low bias | Low bias | Low bias | Low bias | High bias |
| Jaspers, L., 2017 (18) | Low bias | Moderate bias | Moderate bias | Low bias | Low bias | Low bias |
| Ma, W., 2017 (19) | Low bias | Low bias | Moderate bias | Low bias | Low bias | Low bias |
| Ruhunuhewa, I., 2017 (20) | Moderate bias | Low bias | Low bias | Low bias | Low bias | Low bias |
| Gopinath, B., 2016 (21) | Moderate bias | Moderate bias | Moderate bias | Low bias | Low bias | Moderate bias |
| Gopinath, B., 2016 (22) | Moderate bias | Moderate bias | Moderate bias | Low bias | Low bias | Moderate bias |

**STable 7 Risk of bias assessment results domain 6: Statistical analysis and reporting**

| **First author, year (*Ref*)** | 1. **Sufficient presentation of data to assess the adequacy of the analytic strategy** | 1. **Strategy for model building is appropriate and is based on a conceptual framework or model** | 1. **The selected statistical model is adequate for the design of the study** | 1. **There is no selective reporting of results** |
| --- | --- | --- | --- | --- |
| Aalto, U.L., 2023 (1) | Low bias | Low bias | Low bias | Low bias |
| Zhu, X., 2023 (2) | Low bias | Low bias | Low bias | Low bias |
| Oktaviani, L.W., 2022 (3) | Low bias | Low bias | Low bias | Low bias |
| Lee-Bravatti, M. A., 2021 (4) | Low bias | Low bias | Low bias | Low bias |
| Lin, Y. H., 2021 (5) | Low bias | Low bias | Low bias | Low bias |
| Assmann, K.E., 2019 (6) | Low bias | Low bias | Low bias | Low bias |
| Cooney, T.M., 2019 (7) | Low bias | Low bias | Low bias | Low bias |
| James, P., 2019 (8) | Low bias | Low bias | Low bias | Low bias |
| Kim, E.S., 2019 (9) | Low bias | Low bias | Low bias | Low bias |
| Lassale, C., 2019 (10) | Low bias | Low bias | Low bias | Low bias |
| Urtamo, A., 2019 (11) | Low bias | Low bias | Low bias | Low bias |
| Assmann, K.E., 2018 (12) | Low bias | Low bias | Low bias | Low bias |
| Assmann, K.E., 2018 (13) | Low bias | Low bias | Low bias | Low bias |
| Atallah, N., 2018 (14) | Low bias | Low bias | Low bias | Low bias |
| Domènech-Abella, J., 2018 (15) | Low bias | Low bias | Low bias | Low bias |
| Lai, H.T., 2018 (16) | Low bias | Low bias | Low bias | Low bias |
| Rinaldi, J., 2018 (17) | Low bias | Low bias | Low bias | Low bias |
| Jaspers, L., 2017 (18) | Low bias | Low bias | Low bias | Low bias |
| Ma, W., 2017 (19) | Low bias | Low bias | Low bias | Low bias |
| Ruhunuhewa, I., 2017 (20) | Low bias | Low bias | Low bias | Low bias |
| Gopinath, B., 2016 (21) | Low bias | low bias | Low bias | Low bias |
| Gopinath, B., 2016 (22) | Low bias | Low bias | Low bias | Low bias |
|  |  |  |  |  |

**References**

1. Aalto UL, Knuutila M, Lehti T, Jansson A, Kautiainen H, Öhman H*, et al.* Being actively engaged in life in old age: determinants, temporal trends, and prognostic value. Aging Clin Exp Res. 2023.

2. Zhu X, Zhang X, Ding L, Tang Y, Xu A, Yang F*, et al.* Associations of Pain and Sarcopenia with Successful Aging among Older People in China: Evidence from CHARLS. J Nutr Health Aging. 2023;**27**:196-201.

3. Oktaviani LW, Hsu H-C, Chen Y-C. Effects of Health-Related Behaviors and Changes on Successful Aging among Indonesian Older People. International Journal of Environmental Research and Public Health. 2022;**19**:5952.

4. Lee-Bravatti MA, O'Neill HJ, Wurth RC, Sotos-Prieto M, Gao X, Falcon LM*, et al.* Life style behavioral factors and integrative successful aging among puerto ricans living in the Mainland United States. Journals of Gerontology - Series A Biological Sciences and Medical Sciences. 2021;**76**:1108-1116.

5. Lin YH, Chiou JM, Chen TF, Lai LC, Chen JH, Chen YC. The association between metabolic syndrome and successful aging- using an extended definition of successful aging. PLoS ONE. 2021;**16**.

6. Assmann KE, Adjibade M, Adriouch S, Andreeva VA, Julia C, Hercberg S*, et al.* Association of diet quality and physical activity with healthy ageing in the French NutriNet-Santé cohort. Br J Nutr. 2019;**122**:93-102.

7. Cooney TM, Curl AL. Transitioning From Successful Aging: A Life Course Approach. J Aging Health. 2019;**31**:528-551.

8. James P, Kim ES, Kubzansky LD, Zevon ES, Trudel-Fitzgerald C, Grodstein F. Optimism and Healthy Aging in Women. Am J Prev Med. 2019;**56**:116-124.

9. Kim ES, James P, Zevon ES, Trudel-Fitzgerald C, Kubzansky LD, Grodstein F. Optimism and Healthy Aging in Women and Men. Am J Epidemiol. 2019;**188**:1084-1091.

10. Lassale C, Batty GD, Steptoe A, Cadar D, Akbaraly TN, Kivimäki M*, et al.* Association of 10-Year C-Reactive Protein Trajectories With Markers of Healthy Aging: Findings From the English Longitudinal Study of Aging. J Gerontol A Biol Sci Med Sci. 2019;**74**:195-203.

11. Urtamo A, Huohvanainen E, Pitkälä KH, Strandberg TE. Midlife predictors of active and healthy aging (AHA) among older businessmen. Aging Clin Exp Res. 2019;**31**:225-231.

12. Assmann KE, Ruhunuhewa I, Adjibade M, Li Z, Varraso R, Hercberg S*, et al.* The Mediating Role of Overweight and Obesity in the Prospective Association between Overall Dietary Quality and Healthy Aging. Nutrients. 2018;**10**.

13. Assmann KE, Adjibade M, Andreeva VA, Hercberg S, Galan P, Kesse-Guyot E. Association Between Adherence to the Mediterranean Diet at Midlife and Healthy Aging in a Cohort of French Adults. J Gerontol A Biol Sci Med Sci. 2018;**73**:347-354.

14. Atallah N, Adjibade M, Lelong H, Hercberg S, Galan P, Assmann KE*, et al.* How Healthy Lifestyle Factors at Midlife Relate to Healthy Aging. Nutrients. 2018;**10**.

15. Domènech-Abella J, Perales J, Lara E, Moneta MV, Izquierdo A, Rico-Uribe LA*, et al.* Sociodemographic Factors Associated With Changes in Successful Aging in Spain: A Follow-Up Study. J Aging Health. 2018;**30**:1244-1262.

16. Lai HT, de Oliveira Otto MC, Lemaitre RN, McKnight B, Song X, King IB*, et al.* Serial circulating omega 3 polyunsaturated fatty acids and healthy ageing among older adults in the Cardiovascular Health Study: prospective cohort study. Bmj. 2018;**363**:k4067.

17. Rinaldi J, Souza GDC, Camozzato AL, Chaves MLF. Sixteen-year predictors of successful aging from a Southern Brazilian cohort The PALA study. Dement Neuropsychol. 2018;**12**:228-234.

18. Jaspers L, Schoufour JD, Erler NS, Darweesh SK, Portegies ML, Sedaghat S*, et al.* Development of a Healthy Aging Score in the Population-Based Rotterdam Study: Evaluating Age and Sex Differences. J Am Med Dir Assoc. 2017;**18**:276.e271-276.e277.

19. Ma W, Hagan KA, Heianza Y, Sun Q, Rimm EB, Qi L. Adult height, dietary patterns, and healthy aging. Am J Clin Nutr. 2017;**106**:589-596.

20. Ruhunuhewa I, Adjibade M, Andreeva VA, Galan P, Hercberg S, Assmann KE*, et al.* Prospective association between body mass index at midlife and healthy aging among French adults. Obesity (Silver Spring). 2017;**25**:1254-1262.

21. Gopinath B, Flood VM, Kifley A, Louie JC, Mitchell P. Association Between Carbohydrate Nutrition and Successful Aging Over 10 Years. J Gerontol A Biol Sci Med Sci. 2016;**71**:1335-1340.

22. Gopinath B, Russell J, Kifley A, Flood VM, Mitchell P. Adherence to Dietary Guidelines and Successful Aging Over 10 Years. J Gerontol A Biol Sci Med Sci. 2016;**71**:349-355.
